# Myocardial Expression of Pluripotency, Longevity, and Proinflammatory Genes in the Context of Hypercholesterolemia and Statin Treatment

**DOI:** 10.3390/jcm13071994

**Published:** 2024-03-29

**Authors:** Konstantinos S. Mylonas, Michail Peroulis, Emmanouil I. Kapetanakis, Alkistis Kapelouzou

**Affiliations:** 1 Department of Cardiac Surgery, Onassis Cardiac Surgery Center, 356 Leof. Andreas Syngros, 17674 Athens, Greece; 2 Vascular Surgery Unit, Department of Surgery, Faculty of Medicine, University of Ioannina, 45110 Ioannina, Greece; mperoulis@gmail.com; 3 Third Department of Surgery, Attikon University Hospital, National and Kapodistrian University of Athens, 12462 Athens, Greece; emmanouil.kapetanakis@gmail.com; 4 Clinical, Experimental Surgery & Translational Research, Biomedical Research Foundation Academy of Athens, 11527 Athens, Greece; akapel@bioacademy.gr

**Keywords:** myocardium, atheromatosis, klotho, MYD88, pluripotency, stem cell genes, KLF4, NANOG, HOXA5, HIF1α

## Abstract

**Background:** This study sought to assess the effect of statin therapy on myocardial inflammation in a White New Zealand rabbit model of atherogenesis. **Methods:** The mRNA expression levels of pro-inflammatory, pluripotency, and aging-related markers were quantified following a controlled feeding protocol and statin treatments. **Results:** Following high-cholesterol diet induction, we observed significant upregulation in the myocardial mRNA levels of MYD88, NF-κB, chemokines (CCL4, CCL20, and CCR2), IFN-γ, interleukins (IL-1β, IL-2, IL-4, IL-8, IL-10, and IL-18), and novel markers (klotho, KFL4, NANOG, and HIF1α). In contrast, HOXA5 expression was diminished following a hyperlipidemic diet. Both statin treatments significantly influenced the markers studied. Nevertheless, rosuvastatin administration resulted in a greater reduction in MYD88, NF-kB, chemokines (CCL4, CCL20, and CCR2), and interleukins IL-1β, IL-8, KLF4, NANOG, and HIF1α than fluvastatin. Fluvastatin, on the other hand, led to a stronger decrease in IL-4. Downregulation of IL-2 and IL-18 and upregulation of IFNβ and HOXA5 were comparable between the two statins. Notably, rosuvastatin had a stronger effect on the upregulation of klotho and IL-10. **Conclusion:** Overall, statin therapy significantly attenuated inflammatory, pluripotency, and klotho expression in myocardial tissue under atherogenic conditions. Our findings also highlight the differential efficacy of rosuvastatin over fluvastatin in curtailing proatherogenic inflammation, which could have profound implications for the clinical management of cardiovascular disease.

## 1. Introduction

In the past two decades, a robust body of literature has linked vascular inflammation to atheromatosis [1]. Toll-like receptors (TLRs) have been identified as primary contributors to the inflammatory processes that fuel the progression of atherosclerosis [2,3]. The MYD88 (myeloid differentiation primary response protein 88)-dependent pathway is essential for activating all TLRs except TLR3, NF-κB (nuclear factor kappa-light-chain-enhancer of activated B cells), as well as some members of the IL-1 (interleukin-1) receptor family [4]. It also triggers MAPK (mitogen-activated protein kinase), leading to the generation and release of proatherogenic cytokines [5].

Alongside established biomarkers, recent studies are investigating novel atheroprotective agents such as klotho (an anti-aging hormone) and stem cell-related genes including HOXA5 and Krüppel-like factors (KLF). Additional attention is directed towards proatherogenic pluripotency factors such as NANOG and HIF1α [1,6,7,8]. Dysregulation of their expression has been implicated in various aspects of atherogenesis, including increased release of pro-inflammatory cytokines and chemokines, upregulation of adhesion molecules, migration and phenotypic switching of vascular smooth muscle cells (VSMCs), alterations in extracellular matrix, oxidative stress, and the development of neointimal hyperplasia [6,9,10].

Numerous classes of medication have been utilized to mitigate proatherogenic inflammation, with statins demonstrating perhaps the most pronounced efficacy in this regard [11,12,13,14]. Indeed, statins hinder the production of prenylated proteins and disrupt the mevalonate pathway while simultaneously inducing nitric oxide synthase activity and stimulating the expression of KLFs [15,16]. Furthermore, statins diminish endothelial cell activation, downregulate selectin expression, and suppress the release of interferon (IFN)-gamma, thus reducing T-cell activation [17].

Interestingly, a one-month regimen of combined treatment with statins and adherence to a standard diet can effectively inhibit the progression of atherosclerotic changes in the thoracic and abdominal aortic tissues [18]. Our lab previously linked the use of rosuvastatin with several positive pleiotropic effects within atherosclerotic plaques. These effects included diminished lipid cores, reduced macrophage presence within the lipid core, and attenuated micro-angiogenesis compared to a normal diet alone [19].

On the other hand, the impact of hypercholesterolemia on the myocardium itself has not been thoroughly explored [20]. While there is evidence indicating that hypercholesterolemia can trigger myocardial inflammation, apoptosis, and fibrosis even without significant coronary artery disease, the molecular mechanisms driving this phenomenon remain elusive [21,22]. In the present study, we analyzed rabbit myocardial specimens to better understand the transcriptional dynamics of proatherogenic cytokines, pivotal stem cell genes, and the response of klotho in the context of hypercholesterolemia. Additionally, we assessed the impact of statin therapy on these factors to inform future research endeavors.

## 2. Materials and Methods

### 2.1. Animal Experiment

Our translational model has been previously described and validated [18,19,23,24]. The present study utilized 48 male White New Zealand rabbits that were referenced by Mylonas et al. in prior work from our lab [18]. All animals were sourced from Trompetas Breeding Laboratories, Attiki, Greece, and housed within the animal research facility of the Biomedical Research Foundation of the Academy of Athens [18]. After a seven-day habituation phase, the aforementioned rabbits were grouped into eight sets of six animals. A high-cholesterol diet (HCD) comprising 1% cholesterol (product 2RB19 by Mucedola, Milan, Italy) was administered.

The first trial involved three sets receiving HCD for periods of one, two, and three months, designated as G30, G60, and G90, respectively. In the subsequent trial, three groups were initially administered HCD for three months, followed by either standard feed alone for an additional month (G120) or standard feed supplemented with either fluvastatin (GF120) or rosuvastatin (GR120) for an additional month. Two control sets received standard feed for periods of 90 (C90) and 120 (C120) days, correspondingly. Rosuvastatin and fluvastatin were administered orally daily at dosages of 0.7 mg/kg body weight and 2 mg/kg body weight, respectively.

### 2.2. Preparation of Biological Samples

Upon completion of the experimental timeline, the subjects were humanely euthanized using a lethal dose of sodium pentobarbital administered intravenously (100–120 mg/kg). Subsequently, their hearts were excised, purged with diethyl pyrocarbonate (DEPC)-treated water, and immediately preserved at −140 °C for subsequent mRNA (messenger ribonucleic acid) examination.

### 2.3. Target Genes

The following genes were analyzed: MYD88 (myeloid differentiation primary response 88), NF-κB, CCL4 (C–C chemokine ligand), CCL20 (chemokine (C–C motif) ligand 20), CCR2 (C–C chemokine receptor type 2), IFN-β (interferon beta), IFN-γ (interferon gamma), IL-1β (interleukin 1β), IL-2 (interleukin 2), IL-4 (interleukin 4), IL-8 (interleukin 8), IL-10 (interleukin 10), IL-18 (interleukin 18), α-klotho, HOXA5, NANOG, KLF4 (Krüppel-like factor 4), and HIF1α (hypoxia-inducible factor 1-alpha).

### 2.4. Examination of mRNA

For the analysis of mRNA, whole-heart tissue samples were processed to isolate total RNA utilizing the Tri Reagent as per Sigma’s (Saint Louis, MO, USA) guidelines [25]. Primer sequences for each targeted gene (Supplemental Table S1) were determined using Beacon Designer V7.0 software (Premier Biosoft International, Palo Alto, CA, USA).

Additionally, the Trizol kit (Invitrogen, Life Technologies, New York, NY, USA) was employed for total RNA extraction from myocardial samples across all groups. An ultraviolet spectrophotometer (Biomate 3, Thermo Fisher Scientific, Waltham, MA, USA) measured RNA concentrations. A mixture of RNA/hexamer was prepared in 13 µL volume. The RNA (1–10 µL) was blended with DEPC water (3 µL) and denatured at 70 °C for 5 min before being chilled on ice for an equal duration. 

Reverse transcription (RT) then proceeded in 25 µL volume, incorporating RNase inhibitor (50 U/L) (1 µL), reverse transcriptase (M-MLV 200U) (1 µL), dNTP (10 mmol/L; 5 µL), and RT buffer (5 µL) into the denatured RNA (13 µL) and incubated at 37 °C for 60 min. PCR was conducted in 25 µL volume, including 2 µL of complementary DNA (cDNA), 0.5 µL Taq polymerase (5 U/L), 2.5 µL Thermo 10 buffer, 1 µL dNTP (2.5 mmol/L), 17 µL DEPC water, and 1 µL each of the forward and reverse primers. The β-actin gene’s PCR conditions were an initial denaturation at 95 °C for 10 min, followed by a cycle of denaturation at 95 °C for 1 min, annealing at 57 °C for 1 min, extension at 72 °C for 1 min, and then 35 cycles of the same, with a final extension at 72 °C for 5 min and a holding temperature of 10 °C.

Quantitative real-time PCR (qRT-PCR) was performed in 20 µL volume containing 2 µL cDNA, 7 µL DEPC water, 1 µL each of the primers, and 10 µL SYBR-Green, following specific thermal cycling parameters. β-actin was the internal standard for relative gene expression levels, calculated using the ∆∆CT method [25], with results normalized against it and presented relative to controls as described previously [26]. PCR, RT-PCR, and qRT-PCR were all conducted using the PTC-200 Multi Cycler (MJ Research Inc, South San Francisco, CA, USA). The Chromo4 RT-PCR detector gathered gene expression data, which were analyzed with the Opticon Monitor Continuous Fluorescence Detector 3 software (MJ Research Inc., South San Francisco, CA, USA).

### 2.5. Statistical Analysis

Data were presented as means with standard deviations (SD). The differences between means, along with their 95% confidence intervals (CI), were determined. One-way ANOVA was utilized, succeeded by Tukey’s multiple comparison test. Significance was set at a *p*-value less than 0.05. GraphPad Prism 4.03 (Boston, MA, USA) was used for all analyses.

## 3. Results

Our investigation into myocardial cytokine and regulatory gene expression revealed significant patterns both across the various stages of atherogenesis and in response to statin treatment (Table 1 and Supplementary Materials). The expression levels were compared to β-actin and are presented as fold changes (2^−ΔΔCt^).

### 3.1. MYD88

**Figure 1:**
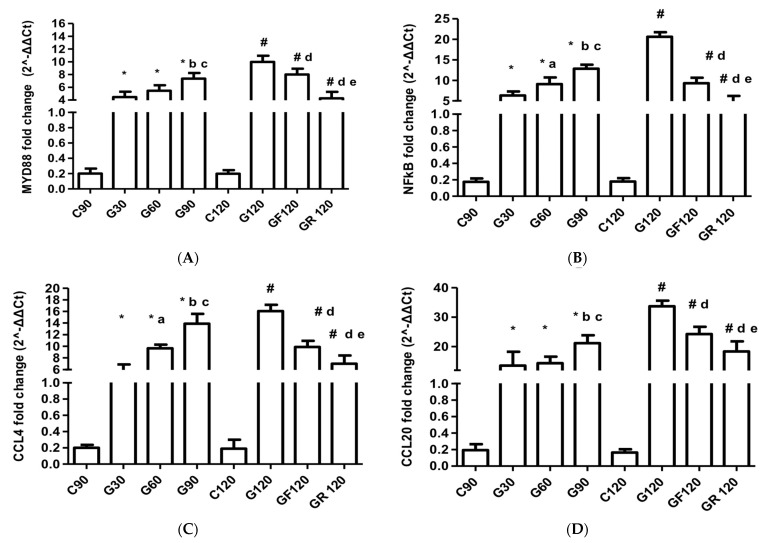

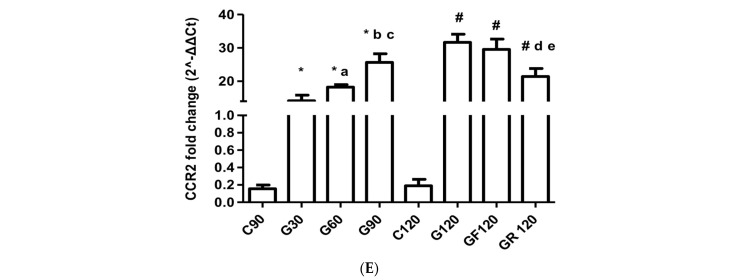
Myocardial mRNA expression of (**A**) MYD88, (**B**) NF-kB, (**C**) CCL4, (**D**) CCL20, and (**E**) CCR2. * C90 vs. G30 or G60 or G90 (*p* < 0.001); a G30 vs. G60; b G30 vs. G90; c G60 vs. G90; # C120 vs. G120 or GF120 or GR120 (*p* < 0.001); d G120 vs. GF120 or GR120; e GF120 vs. GR120.

Myocardial MYD88 mRNA expression varied significantly during atherogenesis and after statin treatment. In the G120 group, the MYD88 mRNA levels increased significantly compared to the G30 group (MD: −5.51, 95% CI [−6.97 to −4.04], *p* < 0.001), G60 group (MD: −4.51, 95% CI [−5.98 to −3.04], *p* < 0.001), and G90 group (MD: −2.42, 95% CI [−4.09 to −1.15], *p* < 0.001). Following the introduction of statins, the GF120 group exhibited a significant reduction in MYD88 expression compared to G120 (MD: 1.97, 95% CI [0.50 to 3.44], *p* < 0.001), with rosuvastatin treatment (GR120) demonstrating an even greater effect (MD: 5.71, 95% CI [4.25 to 7.18], *p* < 0.001, Figure 1A).

### 3.2. NF-kB

The NF-kB mRNA levels displayed a significant increase during the progression of myocardial atherogenesis, with peak levels observed in the G120 group when compared to G30 (MD: −14.31, 95% CI [−16.28 to −12.33], *p* < 0.001), G60 (MD: −11.54, 95% CI [−13.52 to −9.55], *p* < 0.001), and G90 (MD: −7.75, 95% CI [−9.75 to −5.79], *p* < 0.001). Following statin therapy, there was a significant downregulation of NF-kB mRNA in the GF120 group versus the G120 group (MD: 11.30, 95% CI [9.32 to 13.28], *p* < 0.001) and a more pronounced decrease in the GR120 group (MD:15.65, 95% CI [13.67 to 17.63], *p* < 0.001, Figure 1B).

### 3.3. Chemokines

During atherogenesis, the CCL4 mRNA levels escalated significantly by the G120 stage in comparison to G30 (MD: −11.01, 95% CI [−13.17 to −8.84], *p* < 0.001), G60 (MD: −6.41, 95% CI [−8.57 to −4.25], *p* < 0.001), and G90 (MD: −2.15, 95% CI [−4.31 to 0.0005], *p* < 0.001). Treatment with fluvastatin resulted in a significant reduction in CCL4 expression when compared with the G120 group (MD: 6.17, 95% CI [4.01 to 8.33], *p* < 0.001). Rosuvastatin demonstrated an even greater decrease in CCL4 (MD: 9.02, 95% CI [6.86 to 11.18], *p* < 0.001, Figure 1C).

The CCL20 mRNA levels demonstrated a statistically significant increase at the G120 stage compared to the G30 (MD: −20.10, 95% CI [−24.94 to −15.25], *p* < 0.001), G60 (MD: −19.29, 95% CI [−24.14 to −14.45], *p* < 0.001), and G90 groups (MD: −12.54, 95% CI [−17.38 to −7.96], *p* < 0.001). Following statin therapy, the CCL20 mRNA levels significantly decreased in the GF120 group (MD: 9.45, 95% CI [4.61 to −14.30], *p* < 0.001) and more markedly in the GR120 group (MD: 15.37, 95% CI [10.53 to 20.21], *p* < 0.001, Figure 1D).

The CCR2 mRNA levels in myocardial tissue significantly rose at G120 in comparison to G30 (MD: −17.49, 95% CI [−21.14 to −13.83], *p* < 0.001), G60 (MD: −13.42, 95% CI [−17.07 to −9.75], *p* < 0.001), and G90 (MD: −5.98, 95% CI [−9.63 to −2.32], *p* < 0.001). Following statin administration, a significant reduction was observed in the GF120 (MD: 2.08, 95% CI [−1.57 to −5.74], *p* < 0.001) and GR120 groups (MD −10.22, 95% CI [6.56 to 13.88], *p* < 0.001, Figure 1E).

### 3.4. Interferons

**Figure 2:**
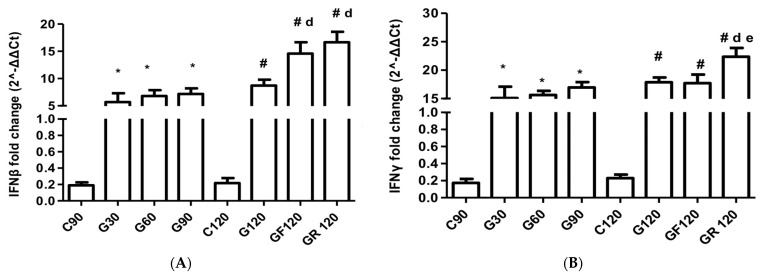
Myocardial mRNA expression of (**A**) IFNβ and (**B**) IFNγ. * C90 vs. G30 or G60 or G90 (*p* < 0.001); a G30 vs. G60; b G30 vs. G90; c G60 vs. G90; # C120 vs. G120 or GF120 or GR120 (*p* < 0.001); d G120 vs. GF120 or GR120; e GF120 vs. GR120.

IFN-β mRNA expression was heightened in the G120 group compared to G30 (MD: −3.03, 95% CI [−5.48 to −0.58], *p* < 0.001) but did not differ significantly compared to G60 and G90. Following statin administration, both the GF120 and GR120 groups demonstrated equally potent significant increases in IFN-β mRNA levels (GF120 group MD: −5.87, 95% CI [−8.31 to −3.42], *p* < 0.001) compared to GR120 (MD: −7.94, 95% CI [−10.39 to −5.49, *p* < 0.001, Figure 2A).

The progression to G120 led to a significant increase in the IFN-γ mRNA levels from G30 (MD: −2.80, 95% CI [−4.95 to −0.65], *p* < 0.001) and G60 (MD: −2.24, 95% CI [−4.39 to −0.09], *p* < 0.001). Only rosuvastatin resulted in significantly increased IFN-γ mRNA compared to G120 (MD: −4.46, 95% CI [−6.61 to −2.31], *p* < 0.001, Figure 2B).

### 3.5. Interleukins

**Figure 3:**
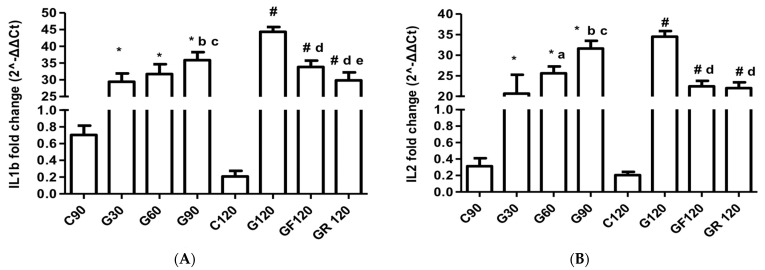

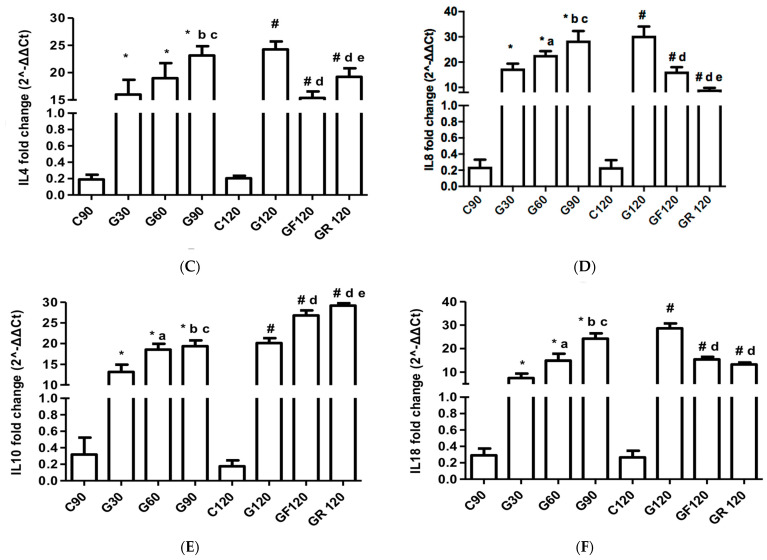
Myocardial mRNA expression of (**A**) IL-1b, (**B**) IL-2, (**C**) IL-4, (**D**) IL-8, (**E**) IL-10, and (**F**) IL-18. * C90 vs. G30 or G60 or G90 (*p* < 0.001); a G30 vs. G60; b G30 vs. G90; c G60 vs. G90; # C120 vs. G120 or GF120 or GR120 (*p* < 0.001); d G120 vs. GF120 or GR120; e GF120 vs. GR120.

The IL-1β levels showed a significant increase at G120 when compared to G30 (MD: −14.94, 95% CI [−18.62 to −11.26], *p* < 0.001), G60 (MD: −12.63, 95% CI [−16.31 to −8.95], *p* < 0.001), and G90 (MD: −8.49, 95% CI [−12.17 to −4.81], *p* < 0.001). Both the GF120 (MD: 10.52, 95% CI [6.83 to 14.20], *p* < 0.001) and GR120 (MD: 14.53, 95% CI [10.85 to 18.21], *p* < 0.001) groups experienced significant reductions in IL-1β mRNA expression, with GR120 showing a more substantial decrease (Figure 3A).

Throughout atherogenesis, the IL-2 mRNA levels were elevated, peaking in the G120 group compared to G30 (MD: −13.84, 95% CI [−17.60 to −10.08], *p* < 0.001) and G60 (MD: −8.90, 95% CI [−12.66 to −5.14], *p* < 0.001). Both statin regimens mediated a reduction in IL-2 expression, with equal potency (MD: 0.44, 95% CI [−3.31 to 4.20], *p* < 0.001, Figure 3B).

IL-4 mRNA expression saw an increase during the progression of atherosclerosis, particularly in the G120 group when juxtaposed with the G30 (MD: −8.26, 95% CI [−11.45 to −5.08], *p* < 0.001) and G60 groups (MD: −5.27, 95% CI [−8.46 to −2.09], *p* < 0.001). Both statins significantly lowered the IL-4 mRNA levels in comparison to G120, especially fluvastatin (MD: −3.87, 95% CI [−7.05 to −0.68], *p* < 0.001) *p* < 0.001, Figure 3C).

IL-8 production was substantially elevated in G120 over G30 (IL-8: MD: −12.94, 95% CI [−16.86 to 2–9.01], *p* < 0.001) and G60 (MD: −7.60, 95% CI [−11.52 to −3.76], *p* < 0.001). Statin therapy markedly reduced IL-8 compared to G120, with rosuvastatin showing a more pronounced reduction as opposed to fluvastatin (IL-8: MD:7.12, 95% CI [3.20 to 11.05], *p* < 0.001, Figure 3D).

IL-10 expression was heightened in the G120 group compared to G30 (MD: −6.96, 95% CI [−9.08 to −4.84], *p* < 0.001) but did not differ compared to G60 and G90. Both statins led to further overexpression of IL-10, albeit rosuvastatin exhibited a significantly stronger effect (MD: −2.37, 95% CI [−4.49 to −0.25], *p* < 0.001, Figure 3E).

The IL-18 mRNA levels increased in the G120 group compared to G30 (MD: −16.68, 95% CI [−19.82 to −13.55], *p* < 0.001), G60 (MD: −13.76, 95% CI [−16.90 to −10.62], *p* < 0.001), G90 (MD: −4.45, 95% CI [−7.54 to −1.32], *p* < 0.001). Both fluvastatin (MD: 13.29, 95% CI [10.16 to 16.43], *p* < 0.001) and rosuvastatin (MD:15.37, 95% CI [12.23 to 18.50], *p* < 0.001) significantly downregulated IL-18 compared to G120, with similar efficacy (Figure 3F).

### 3.6. Klotho

**Figure 4:**
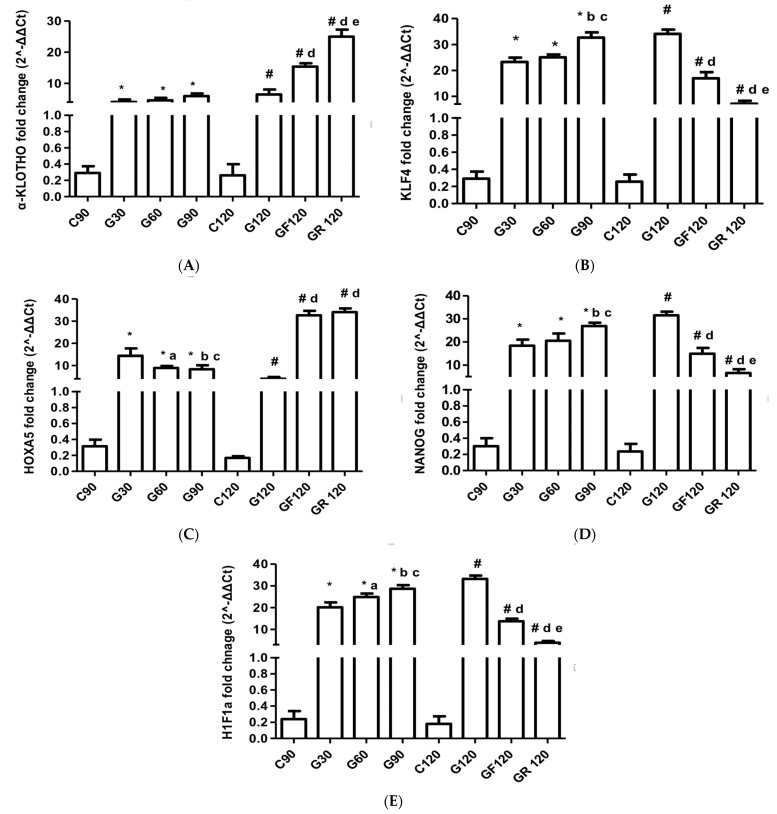
Myocardial mRNA expression of (**A**) klotho, (**B**) KLF4, (**C**) HOXA5, (**D**) NANOG, and (**E**) HIF1α. * C90 vs. G30 or G60 or G90 (*p* < 0.001); a G30 vs. G60; b G30 vs. G90; c G60 vs. G90; # C120 vs. G120 or GF120 or GR120 (*p* < 0.001); d G120 vs. GF120 or GR120; e GF120 vs. GR120.

The klotho mRNA expression in the myocardium at G120 was significantly higher than at G30 (MD: −2.40, 95% CI [−4.53 to −0.27], *p* < 0.001). Following treatment, the GF120 group demonstrated a significant increase in klotho levels (MD: −8.87, 95% CI [−11.00 to −6.74], *p* < 0.001). An even greater upregulation was observed in the GR120 group (MD: −18.48, 95% CI [−20.61 to −16.35], *p* < 0.001, Figure 4A).

### 3.7. Pluripotency Genes

The KLF4 mRNA levels at G120 demonstrated a significant increase from G30 (MD: −10.82, 95% CI [−13.58 to −8.06], *p* < 0.001) and G60 (MD: −9.03, 95% CI [−11.79 to −6.27], *p* < 0.001). Although both statin regimens significantly curtailed KLF4 expression, rosuvastatin administration led to a greater reduction in its production (MD: 9.85, 95% CI [7.09 to 12.61], *p* < 0.001, Figure 4B).

Myocardial HOXA5 production was significantly reduced at G120 compared to G30 (MD: 10.31, 95% CI [7.19 to 13.41], *p* < 0.001), G60 (MD: 3.89, 95% CI [1.78 to 8.00], *p* < 0.001), and G90 (MD: 4.29, 95% CI [1.18 to 7.39], *p* < 0.001). Both fluvastatin (MD: −28.64, 95% CI [−31.74 to −25.53], *p* < 0.001) and rosuvastatin (MD: −30.06, 95% CI [−33.16 to −26.95], *p* < 0.001) substantially upregulated the HOXA5 mRNA levels with similar efficiency (Figure 4C).

NANOG mRNA expression showed significant elevation at G120 compared to G30 (MD: −13.16, 95% CI [−16.76 to −9.56], *p* < 0.001), G60 (MD: −11.04, 95% CI [−14.63 to −7.44], *p* < 0.001), and G90 (MD: −4.64, 95% CI [−8.24 to −1.05], *p* < 0.001). Following statin therapy, the NANOG mRNA levels decreased significantly in the GF120 group (MD: 16.62, 95% CI [13.03 to 20.22], *p* < 0.001) and more markedly in the GR120 group (MD: 31.28, 95% CI [21.28 to 28.47], *p* < 0.001, Figure 4D).

The myocardial HIF1α levels at G120 were significantly upregulated compared to G30 (MD: −13.09, 95% CI [−15.61 to −10.57], *p* < 0.001), G60 (MD: −8.36, 95% CI [−10.88 to −5.84], *p* < 0.001), and G90 (MD: −4.57, 95% CI [−7.09 to −2.05], *p* < 0.001). Statin treatment significantly lowered the HIF1α mRNA levels in GF120 (MD: 19.52, 95% CI [17.00 to 22.04], *p* < 0.001) and even more so in GR120 (MD: 29.36, 95% CI [26.84 to 31.88], *p* < 0.001, Figure 4E).

## 4. Discussion

Our study quantified the mRNA expression of an array of cytokines and regulatory genes, including MYD88, NF-kB, CCL4, CCL20, CCR2, IFN-β, IFN-γ, TNF-α, IL-1b, IL-2, IL-4, IL-8, IL-10, IL-18, klotho, KLF4, HOXA5, NANOG, and HIF1α, against β-actin within myocardial tissue. This expression was tracked through various stages of atherogenesis (G30, G60, G90, and G120) and during treatment with two different statins, fluvastatin (GF) and rosuvastatin (GR). This robust protocol delineated the myocardial inflammatory response following a hypercholesterolemic diet and the differential impact of statin therapy on an experimental animal model.

The myocardial expression of MYD88 and NF-κB showed significant escalation in the G120 phase, thereby affirming the role of innate immunity in advanced atherogenesis [2,8,18,27]. The administration of statins revealed a tiered response: while both fluvastatin and rosuvastatin reduced MYD88 expression, the latter demonstrated a more pronounced effect. Notably, the decrease in MYD88 expression was most significant in the GR120 group compared to the G30, G60, G90, and GF120 groups. NF-κB mirrored this response, suggesting that rosuvastatin may more effectively disrupt the transcriptional machinery that drives proatherogenic inflammation.

Chemokines CCL4, CCL20, and CCR2 are implicated in leukocyte recruitment and endothelial interaction [10,28,29,30]. Their myocardial mRNA levels peaked at G120, corresponding with the heightened inflammatory and immune cell recruitment activity associated with advanced atherogenic stages. Statin therapy not only reversed this trend but did so differentially. Rosuvastatin showed a superior capacity to downregulate these chemokines, particularly CCL20 and CCR2, thus indicating more robust attenuation of monocyte and lymphocyte traffic by this drug.

Myocardial IFN-β levels increased following statin treatment, with no effect difference between the two regimens. IFN-γ increased compared to G120 only with rosuvastatin. This orchestration of interferon immunomodulation suggests an anti-inflammatory statin benefit beyond traditional cholesterol-lowering actions. Prolonged hypercholesterolemia also established an environment rich in both pro- and anti-inflammatory interleukin signaling within myocardial tissue [13,31,32]. Both statin regimens were equally effective in downregulating IL-2 and IL-18. On the other hand, rosuvastatin more effectively curtailed the production of IL-1β and IL-8, whereas fluvastatin proved to be superior in diminishing IL-4 expression. Interestingly, statin administration led to significant upregulation of the anti-inflammatory IL-10, with rosuvastatin seemingly evoking a more robust effect compared to fluvastatin. These findings suggest that statin treatment, and particularly rosuvastatin, may promote the resolution of inflammation and modulation of adaptive immunity within a hyperlipidemic myocardium.

Benchmark laboratory data from other groups are in line with the aforementioned findings [30,33]. Notably, MYD88 deficiency has been shown to result in formation of smaller atherosclerotic plaques. This is attributed to reduced macrophage recruitment and lower levels of chemokines like CCL2 and CCL4 [34]. Furthermore, macrophages from MYD88-deficient mice showed decreased activation, diminished lipid accumulation, and reduction in foam cell formation in response to oxidized low-density lipoprotein (LDL) [35,36]. MYD88 deletion also leads to reduced production of reactive oxygen species, lower secretion of atheroprotective IgM, and decreased expression of inflammatory cytokines and adhesion molecules like IL-1β, IL-6, CXCL1 (C-X-C motif chemokine ligand 1), MCP1 (monocyte chemoattractant protein-1), ICAM1 (intercellular adhesion molecule 1), and VCAM1 (vascular cell adhesion molecule 1), which may reduce T-cell infiltration and atherogenesis [37,38]. Additionally, MYD88-dependent activation in aortic endothelial cells increases PCSK9 (proprotein convertase subtilisin/kexin type 9) expression, suggesting a role for MYD88 in promoting atherosclerosis by affecting endothelial lipid processing [39].

Our analysis also extended into emerging atherosclerosis markers, such as klotho and pluripotency genes [6,7,40,41,42,43,44]. Klotho is an anti-aging hormone that plays a pivotal role in the preservation of endothelial cell integrity [45,46,47]. In vitro studies on human umbilical endothelial cells demonstrate that klotho administration impedes monocyte adhesion through the suppression of TNF α-induced production of adhesion molecules such as ICAM-1 (intercellular adhesion molecule 1) and VCAM-1 (vascular cell adhesion molecule 1) and concurrently diminishes NF-κB activation. Intracellular klotho also inhibits the expression of inflammatory cytokines IL-6 and IL-8, regarding both in vitro and in vivo settings [41]. Beyond its anti-inflammatory effects, klotho exerts antioxidative actions in endothelial and VSMCs, evidenced by reduced expression of Nox2 NADPH (nicotinamide adenine dinucleotide phosphate) oxidase and mitigated angiotensin II-induced superoxide production [48]. It further promotes the expression of antioxidant enzymes, enhancing glutathione levels within VSMCs [49].

The atheroprotective attributes of klotho extend to the prevention of vascular calcification as it hinders the phenotypic transformation of VSMCs into osteoblast-like cells [50]. In the absence of klotho, there is upregulation in the activity of type III cotransporters (PiT-1 and PiT-2), which are known to facilitate phosphate-triggered calcification in VSMCs [50,51,52]. Furthermore, the lack of klotho stimulates the expression of CBFA1 (core-binding factor subunit alpha-1), a transcription factor associated with osteogenesis in VSMCs, thus contributing to increased vascular calcification. Conversely, introducing klotho to VSMCs in a controlled laboratory setting has been observed to reduce phosphate absorption by inhibiting type III cotransporter activity and averting the transformation of VSMCs into an osteochondrogenic state [50].

In our study, the expression of myocardial klotho peaked at G120, suggesting a possible response to cellular stress and a need for repair mechanisms during advanced atherogenesis. On the other hand, in patients with significant coronary artery disease, both serum and coronary wall klotho mRNA levels are notably reduced, and this reduction correlates independently with disease severity [53]. Decreased serum klotho levels have also been linked with greater carotid artery intima-media thickness and more advanced peripheral artery disease [54]. The observed variations in klotho expression across different tissues imply tissue-specific tropism to hyperlipidemic conditions.

We recently conveyed that colchicine-based treatment can upregulate klotho levels in atherosclerotic aortic tissue [6]. While colchicine remains the sole FDA-approved immunomodulator targeting proatherogenic inflammation with effects on klotho levels (among other molecules) [55], our research presents a novel therapeutic pathway for consideration. In the present study, klotho expression was markedly enhanced following treatment with both rosuvastatin and fluvastatin. That said, the former was significantly more effective in upregulating klotho and as such in modulating anti-aging and proatherogenic pathways.

KLF4 overexpression contributes to vascular homeostasis via upregulation of endothelial nitric oxide synthase, vascular endothelial-cadherin (VE-cadherin), and connexin 40 [56,57,58,59,60]. KLF4 also inhibits the expression of adhesion molecules and facilitates the transition of macrophages from a pro-inflammatory to an anti-inflammatory phenotype [61,62]. In our study, the KLF4 mRNA levels at G120 were substantially higher compared to G30 and G60. Statin therapy, especially in the context of rosuvastatin, markedly reduced KLF4 production. As observed with colchicine, this effect likely stemmed from a diminished proatherogenic inflammatory response rather than from the drug directly engaging with the KLF4 gene [63].

Consumption of a hypercholesterolemic diet results in notable suppression of the atheroprotective gene HOXA5 in the abdominal aorta [6]. HOXA5 downregulation also correlates with neo-intimal hyperplasia and aberrant angiogenesis [6,9,64]. This facilitates a proatherogenic milieu, characterized by modifications in the extracellular matrix and integrin dynamics, as well as a phenotypic shift in macrophages and VSMCs towards an inflammatory M1 phenotype [43]. During the hyperlipidemic phase of our experiment, the myocardial expression of HOXA5 was markedly reduced. Statin treatment significantly enhanced the synthesis of HOXA5, thereby reinforcing their role in modulating pluripotency disarray during the development of atherosclerosis.

NANOG, a key marker in primordial cellular regulation and pluripotency, has been implicated in vascular pathology by promoting osteopontin expression, VSMC phenotypic changes, cellular proliferation, migration, and survival, as well as by disrupting cell–cell adhesion through VE-cadherin displacement [65,66,67,68]. NANOG upregulation has also been shown to promote neointimal hyperplasia and thoracic aortic atherogenesis [6,9]. Our findings contribute to this body of literature by demonstrating that myocardial NANOG expression is upregulated in response to hyperlipidemic stimuli and is reduced following statin therapy.

HIF1α, a key mediator under hypoxic conditions [69,70], was progressively upregulated during the various stages of hypercholesterolemia, which could reflect an adaptive mechanism to ischemic conditions within the atherosclerotic myocardium [71,72,73]. Similar findings have been reported in abdominal aortic tissue [6]. HIF1α contributes to the development of atheromatosis by fostering intraplaque angiogenesis, stimulating the production of adhesion molecules, and upregulating glucose metabolism enzymes as well as KLF4 [71,72,73]. The observed reduction in HIF1α expression following both fluvastatin and rosuvastatin treatment may indicate an improvement in myocardial oxygenation or a direct effect on the aforementioned intricate signaling pathway.

Similar to observations in thoracic and abdominal aortic tissue [18], the present study in the myocardium indicates that rosuvastatin has a stronger effect in lowering the expression of inflammatory markers than fluvastatin. This suggests that, in the context of myocardial inflammation, rosuvastatin may be the more potent agent in mitigating proatherogenic inflammatory processes.

Our study is subject to certain limitations. First, the sample size for each test group was limited to abide by the 3R (Replacement, Reduction, and Refinement) principles of ethical animal research [74]. Second, we analyzed whole-tissue samples and as such cannot comment on cell-type-level or blood vessel distinctions. Third, due to resource scarcity, our study was focused on transcriptional data, and we did not delve into protein-level analyses for any of the target molecules, including HIF1α. Lastly, our study was not equipped to explore the precise pathways through which hypercholesterolemia and statins affect cytokine, stem cell gene, and klotho expression. Future research endeavors by our team will probe into the detailed mechanisms impacting the myocardium in the setting of atheromatosis and their influence on the production of proinflammatory biomarkers. We also intend to compare rosuvastatin with other potent medication within the same drug class, including atorvastatin and pitavastatin.

## 5. Conclusions

In conclusion, our research has successfully mapped out the diverse and stage-dependent expression patterns of key mediators in proatherogenic inflammation within the myocardium. We have also demonstrated the significant regulatory impact of statins, particularly noting the heightened efficacy of rosuvastatin. This work enriches our comprehension of statin capabilities and underscores the critical roles of pluripotency genes and klotho in cardiovascular disease progression.

## Figures and Tables

**Table 1:** Statistical analysis of myocardial mRNA expression for each biomarker in all study groups.

Genes		C90	G30	G60	G90	C120	G120	GF120	GR 120
**MYD88**	Mean	0.20	4.47	5.46	7.36	0.19	9.98	8.01	4.26
	SD	0.06	0.84	0.84	0.88	0.04	0.96	0.91	1.02
**NF-kB**	Mean	0.17	6.30	9.07	12.84	0.17	20.61	9.31	4.96
	SD	0.04	0.96	1.64	0.99	0.04	1.11	1.31	1.25
**CCL4**	Mean	0.20	5.04	9.63	13.89	0.19	16.05	9.87	7.02
	SD	0.03	1.86	0.64	1.68	0.10	1.09	1.06	1.37
**CCL20**	Mean	0.19	13.60	14.40	21.15	0.16	33.69	24.23	18.32
	SD	0.07	4.63	2.18	2.68	0.03	1.91	2.47	3.44
**CCR2**	Mean	0.15	14.15	18.22	25.65	0.19	31.63	29.55	21.41
	SD	0.04	1.68	0.77	2.56	0.07	2.44	3.09	2.41
**IFN-β**	Mean	0.18	5.67	6.79	7.18	0.217	8.71	14.59	16.66
	SD	0.03	1.64	1.08	1.01	0.06	1.07	2.08	1.91
**IFN-γ**	Mean	0.17	15.09	15.65	16.95	0.22	17.89	17.73	22.36
	SD	0.04	2.01	0.72	0.96	0.04	0.82	1.50	1.54
**IL-1b**	Mean	0.70	29.43	31.74	35.88	0.20	44.37	33.85	29.84
	SD	0.11	2.48	2.92	2.40	0.06	1.42	1.88	2.39
**IL-2**	Mean	0.31	20.66	25.60	31.62	0.20	34.50	22.44	22.00
	SD	0.09	4.59	1.67	1.89	0.04	1.38	1.34	1.40
**IL-4**	Mean	0.19	15.99	18.98	23.14	0.20	24.26	15.37	19.24
	SD	0.05	2.69	2.75	1.71	0.03	1.46	1.19	1.56
**IL-8**	Mean	0.24	17.51	22.85	28.53	0.23	30.45	16.30	9.17
	SD	0.08	1.85	1.47	3.75	0.08	3.61	1.72	0.64
**IL-10**	Mean	0.31	13.15	18.51	19.36	0.176	20.11	26.80	29.17
	SD	0.20	1.79	1.40	1.39	0.07	1.19	1.24	0.64
**IL-18**	Mean	0.29	7.50	14.89	24.19	0.26	28.65	15.35	13.28
	SD	0.08	1.85	2.87	2.30	0.08	2.06	1.07	0.82
**α-Klotho**	Mean	0.23	4.08	4.60	5.96	0.26	6.48	15.35	24.96
	SD	0.09	0.77	0.78	0.80	0.13	1.57	1.07	2.27
**KLF4**	Mean	0.25	23.25	25.04	32.66	0.25	34.08	16.97	7.12
	SD	0.10	1.68	1.06	2.00	0.08	1.68	2.36	1.17
**HOXA5**	Mean	0.31	14.33	8.91	8.31	0.16	4.02	32.66	34.08
	SD	0.08	3.32	0.91	1.80	0.02	0.82	2.00	1.68
**NANOG**	Mean	0.30	18.35	20.47	26.87	0.23	31.51	14.89	6.63
	SD	0.09	2.64	3.14	1.46	0.09	1.58	2.50	1.60
**HIF1α**	Mean	0.24	20.17	24.90	28.69	0.18	33.26	13.74	3.90
	SD	0.09	2.25	1.62	1.67	0.09	1.49	1.23	0.79

Myeloid differentiation primary response 88 (MYD88); nuclear factor kappa-light-chain-enhancer of activated B cells (NF-κB); C–C chemokine ligand (CCL4); chemokine (C–C motif) ligand 20 (CCL20); C–C chemokine receptor type 2 (CCR2); interferon beta (IFN-β); interferon gamma (IFN-γ); interleukin 1β (IL-1β); interleukin 2 (IL-2); interleukin 4 (IL-4); interleukin 8 (IL-8); interleukin 10 (IL-10); interleukin 18 (IL-18); Krüppel-like factor 4 (KLF4); hypoxia-inducible factor 1-alpha (HIF1α). G30: group subjected to a high-cholesterol diet (HCD) for one month; G60: group receiving HCD for two months; G90: group on HCD for three months; G120: group fed HCD for three months followed by standard feed for one month; GF120: group administered HCD for three months, then treated with fluvastatin for one month; GR120: group on HCD for three months, subsequently receiving rosuvastatin for one month. C90: control group on standard feed for 90 days; C120: control group receiving standard feed for 120 days.

## Data Availability

Raw data may be provided by the authors upon request.

## Supplementary Materials

The following supporting information can be downloaded at: https://www.mdpi.com/article/10.3390/jcm13071994/s1.
