# Supplementary material for: Myocardial Expression of Pluripotency, Longevity, and Proinflammatory Genes in the Context of Hypercholesterolemia and Statin Treatment
Source: J Clin Med. 2024 Mar 29;13(7):1994. doi: 10.3390/jcm13071994 (PMC11012955; doi:10.3390/jcm13071994)
Supplement: Supplementary file 1 [file jcm-13-01994-s001.zip › Supplemental Material.pdf]

Parameter	
Table Analyzed	MYD88

One-way analysis of variance	
P value	P<0.0001
P value summary	***
Are means signif. different? (P < 0.05)	Yes
Number of groups	8
F	117,5
R squared	0,9536

Bartlett's test for equal variances	
Bartlett's statistic (corrected)	41,80
P value	P<0.0001
P value summary	***
Do the variances differ signif. (P < 0.05	Yes

ANOVA Table	SS	df	MS
Treatment (between columns)	519,5	7	74,22
Residual (within columns)	25,27	40	0,6317
Total	544,8	47	

Tukey's Multiple Comparison Test	Mean Diff.	q	Significant	Summary	95% CI of diff
C90 vs G30	-4,272	13,16	Yes	***	-5.738 to -2.805
C90 vs G60	-5,268	16,24	Yes	***	-6.735 to -3.801
C90 vs G90	-7,160	22,07	Yes	***	-8.627 to -5.693
C90 vs C120	0,001167	0,003596	No	ns	-1.466 to 1.468
C90 vs G120	-9,783	30,15	Yes	***	-11.25 to -8.316
C90 vs GF120	-7,810	24,07	Yes	***	-9.277 to -6.343
C90 vs GR 120	-4,067	12,53	Yes	***	-5.533 to -2.600
G30 vs G60	-0,9967	3,072	No	ns	-2.464 to 0.4702
G30 vs G90	-2,888	8,902	Yes	***	-4.355 to -1.421
G30 vs C120	4,273	13,17	Yes	***	2.806 to 5.740
G30 vs G120	-5,512	16,99	Yes	***	-6.979 to -4.045
G30 vs GF120	-3,538	10,91	Yes	***	-5.005 to -2.071
G30 vs GR 120	0,2050	0,6318	No	ns	-1.262 to 1.672
G60 vs G90	-1,892	5,830	Yes	**	-3.359 to -0.4248
G60 vs C120	5,269	16,24	Yes	***	3.802 to 6.736
G60 vs G120	-4,515	13,92	Yes	***	-5.982 to -3.048
G60 vs GF120	-2,542	7,833	Yes	***	-4.009 to -1.075
G60 vs GR 120	1,202	3,704	No	ns	-0.2652 to 2.669
G90 vs C120	7,161	22,07	Yes	***	5.694 to 8.628
G90 vs G120	-2,623	8,085	Yes	***	-4.090 to -1.156
G90 vs GF120	-0,6500	2,003	No	ns	-2.117 to 0.8169
G90 vs GR 120	3,093	9,534	Yes	***	1.626 to 4.560
C120 vs G120	-9,784	30,16	Yes	***	-11.25 to -8.317
C120 vs GF120	-7,811	24,07	Yes	***	-9.278 to -6.344
C120 vs GR 120	-4,068	12,54	Yes	***	-5.535 to -2.601
G120 vs GF120	1,973	6,082	Yes	**	0.5064 to 3.440
G120 vs GR 120	5,717	17,62	Yes	***	4.250 to 7.184

GF120 vs GR 120	3,743	11,54	Yes	***	2.276 to 5.210

Parameter					
Table Analyzed	NFKB				
One-way analysis of variance					
P value	P<0.0001				
P value summary	***				
Are means signif. different? (P < 0.05)	Yes				
Number of groups	8				
F	238,4				
R squared	0,9766				
Bartlett's test for equal variances					
Bartlett's statistic (corrected)	54,62				
P value	P<0.0001				
P value summary	***				
Do the variances differ signif. (P < 0.0	Yes				
ANOVA Table	SS	df	MS		
Treatment (between columns)	1919	7	274,1		
Residual (within columns)	45,99	40	1,150		
Total	1965	47			
Tukey's Multiple Comparison Test	Mean Diff.	q	Significant	Summary	95% CI of diff
C90 vs G30	-6,131	14,00	Yes	***	-8.110 to -4.151
C90 vs G60	-8,897	20,32	Yes	***	-10.88 to -6.918
C90 vs G90	-12,66	28,92	Yes	***	-14.64 to -10.68
C90 vs C120	-0,002333	0,005330	No	ns	-1.982 to 1.977
C90 vs G120	-20,44	46,68	Yes	***	-22.41 to -18.46
C90 vs GF120	-9,134	20,86	Yes	***	-11.11 to -7.155
C90 vs GR 120	-4,784	10,93	Yes	***	-6.763 to -2.805
G30 vs G60	-2,767	6,320	Yes	**	-4.746 to -0.7875
G30 vs G90	-6,530	14,92	Yes	***	-8.509 to -4.551
G30 vs C120	6,128	14,00	Yes	***	4.149 to 8.107
G30 vs G120	-14,31	32,68	Yes	***	-16.28 to -12.33
G30 vs GF120	-3,003	6,860	Yes	***	-4.983 to -1.024
G30 vs GR 120	1,347	3,076	No	ns	-0.6325 to 3.326
G60 vs G90	-3,763	8,597	Yes	***	-5.743 to -1.784
G60 vs C120	8,895	20,32	Yes	***	6.916 to 10.87
G60 vs G120	-11,54	26,36	Yes	***	-13.52 to -9.559
G60 vs GF120	-0,2367	0,5406	No	ns	-2.216 to 1.743
G60 vs GR 120	4,113	9,396	Yes	***	2.134 to 6.093
G90 vs C120	12,66	28,91	Yes	***	10.68 to 14.64
G90 vs G120	-7,775	17,76	Yes	***	-9.754 to -5.796
G90 vs GF120	3,527	8,056	Yes	***	1.547 to 5.506
G90 vs GR 120	7,877	17,99	Yes	***	5.897 to 9.856
C120 vs G120	-20,43	46,68	Yes	***	-22.41 to -18.45
C120 vs GF120	-9,131	20,86	Yes	***	-11.11 to -7.152
C120 vs GR 120	-4,782	10,92	Yes	***	-6.761 to -2.802
G120 vs GF120	11,30	25,82	Yes	***	9.322 to 13.28
G120 vs GR 120	15,65	35,75	Yes	***	13.67 to 17.63

GF120 vs GR 120	4,350	9,937	Yes	***	2.371 to 6.329

Parameter					
Table Analyzed	CCL4				
One-way analysis of variance					
P value	P<0.0001				
P value summary	***				
Are means signif. different?	Yes				
Number of groups	8				
F	147,9				
R squared	0,9628				
Bartlett's test for equal variances					
Bartlett's statistic (corrected	52,36				
P value	P<0.0001				
P value summary	***				
Do the variances differ sign	Yes				
ANOVA Table	SS	df	MS		
Treatment (between colum	1420	7	202,8		
Residual (within columns)	54,88	40	1,372		
Total	1475	47			
Tukey's Multiple Compariso	Mean Diff.	q	Significant	Summary	95% CI of diff
C90 vs G30	-4,838	10,12	Yes	***	-7.000 to -2.677
C90 vs G60	-9,433	19,73	Yes	***	-11.60 to -7.272
C90 vs G90	-13,69	28,63	Yes	***	-15.85 to -11.53
C90 vs C120	0,01083	0,02266	No	ns	-2.151 to 2.173
C90 vs G120	-15,85	33,14	Yes	***	-18.01 to -13.68
C90 vs GF120	-9,677	20,24	Yes	***	-11.84 to -7.515
C90 vs GR 120	-6,825	14,27	Yes	***	-8.987 to -4.663
G30 vs G60	-4,595	9,610	Yes	***	-6.757 to -2.433
G30 vs G90	-8,852	18,51	Yes	***	-11.01 to -6.690
G30 vs C120	4,849	10,14	Yes	***	2.687 to 7.011
G30 vs G120	-11,01	23,02	Yes	***	-13.17 to -8.847
G30 vs GF120	-4,838	10,12	Yes	***	-7.000 to -2.677
G30 vs GR 120	-1,987	4,155	No	ns	-4.148 to 0.1752
G60 vs G90	-4,257	8,902	Yes	***	-6.418 to -2.095
G60 vs C120	9,444	19,75	Yes	***	7.282 to 11.61
G60 vs G120	-6,413	13,41	Yes	***	-8.575 to -4.252
G60 vs GF120	-0,2433	0,5089	No	ns	-2.405 to 1.918
G60 vs GR 120	2,608	5,455	Yes	**	0.4465 to 4.770
G90 vs C120	13,70	28,65	Yes	***	11.54 to 15.86
G90 vs G120	-2,157	4,510	No	ns	-4.318 to 0.005151
G90 vs GF120	4,013	8,393	Yes	***	1.852 to 6.175
G90 vs GR 120	6,865	14,36	Yes	***	4.703 to 9.027
C120 vs G120	-15,86	33,16	Yes	***	-18.02 to -13.70
C120 vs GF120	-9,688	20,26	Yes	***	-11.85 to -7.526
C120 vs GR 120	-6,836	14,30	Yes	***	-8.998 to -4.674
G120 vs GF120	6,170	12,90	Yes	***	4.008 to 8.332
G120 vs GR 120	9,022	18,87	Yes	***	6.860 to 11.18

GF120 vs GR 120	2,852	5,964	Yes	**	0.6898 to 5.013

Parameter	
Table Analyzed	CCL20

One-way analysis of variance	
P value	P<0.0001
P value summary	***
Are means signif. different?	Yes
Number of groups	8
F	114,6
R squared	0,9525

Bartlett's test for equal variances	
Bartlett's statistic (corrected	69,85
P value	P<0.0001
P value summary	***
Do the variances differ sign	Yes

ANOVA Table	SS	df	MS
Treatment (between column	5526	7	789,4
Residual (within columns)	275,4	40	6,886
Total	5801	47	

Tukey's Multiple Compariso	Mean Diff.	q	Significant	Summary	95% CI of diff
C90 vs G30	-13,40	12,51	Yes	***	-18.25 to -8.559
C90 vs G60	-14,20	13,26	Yes	***	-19.05 to -9.361
C90 vs G90	-20,96	19,56	Yes	***	-25.80 to -16.12
C90 vs C120	0,0290	0,02707	No	ns	-4.814 to 4.872
C90 vs G120	-33,50	31,27	Yes	***	-38.34 to -28.65
C90 vs GF120	-24,04	22,44	Yes	***	-28.88 to -19.20
C90 vs GR 120	-18,13	16,92	Yes	***	-22.97 to -13.28
G30 vs G60	-0,8017	0,7483	No	ns	-5.645 to 4.042
G30 vs G90	-7,557	7,054	Yes	***	-12.40 to -2.713
G30 vs C120	13,43	12,54	Yes	***	8.588 to 18.27
G30 vs G120	-20,10	18,76	Yes	***	-24.94 to -15.25
G30 vs GF120	-10,64	9,929	Yes	***	-15.48 to -5.793
G30 vs GR 120	-4,725	4,411	No	ns	-9.568 to 0.1183
G60 vs G90	-6,755	6,305	Yes	**	-11.60 to -1.912
G60 vs C120	14,23	13,29	Yes	***	9.390 to 19.08
G60 vs G120	-19,29	18,01	Yes	***	-24.14 to -14.45
G60 vs GF120	-9,835	9,180	Yes	***	-14.68 to -4.992
G60 vs GR 120	-3,923	3,662	No	ns	-8.767 to 0.9200
G90 vs C120	20,99	19,59	Yes	***	16.14 to 25.83
G90 vs G120	-12,54	11,70	Yes	***	-17.38 to -7.695
G90 vs GF120	-3,080	2,875	No	ns	-7.923 to 1.763
G90 vs GR 120	2,832	2,643	No	ns	-2.012 to 7.675
C120 vs G120	-33,53	31,30	Yes	***	-38.37 to -28.68
C120 vs GF120	-24,07	22,47	Yes	***	-28.91 to -19.22
C120 vs GR 120	-18,16	16,95	Yes	***	-23.00 to -13.31
G120 vs GF120	9,458	8,829	Yes	***	4.615 to 14.30
G120 vs GR 120	15,37	14,35	Yes	***	10.53 to 20.21

GF120 vs GR 120	5,912	5,518	Yes	**	1.068 to 10.75

Parameter			
Table Analyzed	CCR2		
One-way analysis of variance			
P value	P<0.0001		
P value summary	***		
Are means signif. different?	Yes		
Number of groups	8		
F	226,9		
R squared	0,9754		
Bartlett's test for equal variances			
Bartlett's statistic (corrected	66,38		
P value	P<0.0001		
P value summary	***		
Do the variances differ signi	Yes		
ANOVA Table	SS	df	MS
Treatment (between column	6232	7	890,4
Residual (within columns)	157,0	40	3,925
Total	6389	47	

Tukey's Multiple Compariso	Mean Diff.	q	Significant	Summary	95% CI of diff
C90 vs G30	-13,99	17,30	Yes	***	-17.65 to -10.33
C90 vs G60	-18,06	22,33	Yes	***	-21.72 to -14.41
C90 vs G90	-25,50	31,52	Yes	***	-29.15 to -21.84
C90 vs C120	-0,0350	0,04328	No	ns	-3.691 to 3.621
C90 vs G120	-31,48	38,92	Yes	***	-35.13 to -27.82
C90 vs GF120	-29,39	36,34	Yes	***	-33.05 to -25.74
C90 vs GR 120	-21,26	26,28	Yes	***	-24.91 to -17.60
G30 vs G60	-4,073	5,037	Yes	*	-7.730 to -0.4170
G30 vs G90	-11,51	14,23	Yes	***	-15.16 to -7.850
G30 vs C120	13,95	17,25	Yes	***	10.30 to 17.61
G30 vs G120	-17,49	21,62	Yes	***	-21.14 to -13.83
G30 vs GF120	-15,40	19,05	Yes	***	-19.06 to -11.75
G30 vs GR 120	-7,267	8,985	Yes	***	-10.92 to -3.610
G60 vs G90	-7,433	9,191	Yes	***	-11.09 to -3.777
G60 vs C120	18,03	22,29	Yes	***	14.37 to 21.68
G60 vs G120	-13,42	16,59	Yes	***	-17.07 to -9.759
G60 vs GF120	-11,33	14,01	Yes	***	-14.99 to -7.674
G60 vs GR 120	-3,193	3,948	No	ns	-6.850 to 0.4630
G90 vs C120	25,46	31,48	Yes	***	21.80 to 29.12
G90 vs G120	-5,982	7,396	Yes	***	-9.638 to -2.325
G90 vs GF120	-3,897	4,818	Yes	*	-7.553 to -0.2403
G90 vs GR 120	4,240	5,243	Yes	*	0.5836 to 7.896
C120 vs G120	-31,44	38,88	Yes	***	-35.10 to -27.79
C120 vs GF120	-29,36	36,30	Yes	***	-33.01 to -25.70
C120 vs GR 120	-21,22	26,24	Yes	***	-24.88 to -17.56
G120 vs GF120	2,085	2,578	No	ns	-1.571 to 5.741
G120 vs GR 120	10,22	12,64	Yes	***	6.565 to 13.88

GF120 vs GR 120	8,137	10,06	Yes	***	4.480 to 11.79

Parameter	
Table Analyzed	IFNb

One-way analysis of variance	
P value	P<0.0001
P value summary	***
Are means signif. differe	Yes
Number of groups	8
F	119,9
R squared	0,9545

Bartlett's test for equal variances	
Bartlett's statistic (correc	58,93
P value	P<0.0001
P value summary	***
Do the variances differ s	Yes

ANOVA Table	SS	df	MS
Treatment (between col	1476	7	210,8
Residual (within column	70,35	40	1,759
Total	1546	47	

Tukey's Multiple Compa	Mean Diff.	q	Significant	Summary	95% CI of diff
C90 vs G30	-5,489	10,14	Yes	***	-7.936 to -3.041
C90 vs G60	-6,604	12,20	Yes	***	-9.051 to -4.156
C90 vs G90	-6,995	12,92	Yes	***	-9.443 to -4.548
C90 vs C120	-0,0275	0,05079	No	ns	-2.475 to 2.420
C90 vs G120	-8,525	15,75	Yes	***	-10.97 to -6.078
C90 vs GF120	-14,40	26,59	Yes	***	-16.84 to -11.95
C90 vs GR 120	-16,47	30,42	Yes	***	-18.92 to -14.02
G30 vs G60	-1,115	2,059	No	ns	-3.563 to 1.333
G30 vs G90	-1,507	2,783	No	ns	-3.954 to 0.9410
G30 vs C120	5,461	10,09	Yes	***	3.014 to 7.909
G30 vs G120	-3,037	5,609	Yes	**	-5.484 to -0.5890
G30 vs GF120	-8,908	16,45	Yes	***	-11.36 to -6.461
G30 vs GR 120	-10,98	20,28	Yes	***	-13.43 to -8.532
G60 vs G90	-0,3917	0,7234	No	ns	-2.839 to 2.056
G60 vs C120	6,576	12,15	Yes	***	4.129 to 9.024
G60 vs G120	-1,922	3,549	No	ns	-4.369 to 0.5260
G60 vs GF120	-7,793	14,39	Yes	***	-10.24 to -5.346
G60 vs GR 120	-9,865	18,22	Yes	***	-12.31 to -7.417
G90 vs C120	6,968	12,87	Yes	***	4.520 to 9.415
G90 vs G120	-1,530	2,826	No	ns	-3.978 to 0.9176
G90 vs GF120	-7,402	13,67	Yes	***	-9.849 to -4.954
G90 vs GR 120	-9,473	17,50	Yes	***	-11.92 to -7.026
C120 vs G120	-8,498	15,70	Yes	***	-10.95 to -6.050
C120 vs GF120	-14,37	26,54	Yes	***	-16.82 to -11.92
C120 vs GR 120	-16,44	30,37	Yes	***	-18.89 to -13.99
G120 vs GF120	-5,872	10,85	Yes	***	-8.319 to -3.424
G120 vs GR 120	-7,943	14,67	Yes	***	-10.39 to -5.496

GF120 vs GR 120	-2,072	3,827	No	ns	-4.519 to 0.3760

Parameter	
Table Analyzed	IFNa

One-way analysis of variance	
P value	P<0.0001
P value summary	***
Are means signif. different? (P <	Yes
Number of groups	8
F	308,4
R squared	0,9818

Bartlett's test for equal variances	
Bartlett's statistic (corrected)	59,93
P value	P<0.0001
P value summary	***
Do the variances differ signif. (P	Yes

ANOVA Table	SS	df	MS
Treatment (between columns)	2927	7	418,1
Residual (within columns)	54,23	40	1,356
Total	2981	47	

Tukey's Multiple Comparison Te	Mean Diff.	q	Significant	Summary	95% CI of diff
C90 vs G30	-14,91	31,38	Yes	***	-17.06 to -12.77
C90 vs G60	-15,47	32,55	Yes	***	-17.62 to -13.32
C90 vs G90	-16,78	35,30	Yes	***	-18.93 to -14.63
C90 vs C120	-0,05600	0,1178	No	ns	-2.205 to 2.093
C90 vs G120	-17,72	37,27	Yes	***	-19.87 to -15.57
C90 vs GF120	-17,55	36,93	Yes	***	-19.70 to -15.40
C90 vs GR 120	-22,18	46,66	Yes	***	-24.33 to -20.03
G30 vs G60	-0,5583	1,175	No	ns	-2.707 to 1.591
G30 vs G90	-1,863	3,920	No	ns	-4.012 to 0.2857
G30 vs C120	14,86	31,26	Yes	***	12.71 to 17.01
G30 vs G120	-2,803	5,897	Yes	**	-4.952 to -0.6543
G30 vs GF120	-2,638	5,550	Yes	**	-4.787 to -0.4893
G30 vs GR 120	-7,267	15,29	Yes	***	-9.416 to -5.118
G60 vs G90	-1,305	2,745	No	ns	-3.454 to 0.8441
G60 vs C120	15,42	32,43	Yes	***	13.27 to 17.57
G60 vs G120	-2,245	4,723	Yes	*	-4.394 to -0.09592
G60 vs GF120	-2,080	4,376	No	ns	-4.229 to 0.06907
G60 vs GR 120	-6,708	14,11	Yes	***	-8.857 to -4.559
G90 vs C120	16,72	35,18	Yes	***	14.57 to 18.87
G90 vs G120	-0,9400	1,977	No	ns	-3.089 to 1.209
G90 vs GF120	-0,7750	1,630	No	ns	-2.924 to 1.374
G90 vs GR 120	-5,403	11,37	Yes	***	-7.552 to -3.254
C120 vs G120	-17,66	37,16	Yes	***	-19.81 to -15.51
C120 vs GF120	-17,50	36,81	Yes	***	-19.65 to -15.35
C120 vs GR 120	-22,13	46,54	Yes	***	-24.27 to -19.98
G120 vs GF120	0,1650	0,3471	No	ns	-1.984 to 2.314
G120 vs GR 120	-4,463	9,389	Yes	***	-6.612 to -2.314

GF120 vs GR 120	-4,628	9,737	Yes	***	-6.777 to -2.479

Parameter					
Table Analyzed	IL1b				
One-way analysis of variance					
P value	P<0.0001				
P value summary	***				
Are means signif. different? (P <	Yes				
Number of groups	8				
F	401,2				
R squared	0,9860				
Bartlett's test for equal variances					
Bartlett's statistic (corrected)	53,00				
P value	P<0.0001				
P value summary	***				
Do the variances differ signif. (P	Yes				
ANOVA Table	SS	df	MS		
Treatment (between columns)	11160	7	1595		
Residual (within columns)	159,0	40	3,975		
Total	11320	47			
Tukey's Multiple Comparison Te	Mean Diff.	q	Significant	Summary	95% CI of diff
C90 vs G30	-28,72	35,29	Yes	***	-32.40 to -25.04
C90 vs G60	-31,03	38,13	Yes	***	-34.71 to -27.35
C90 vs G90	-35,17	43,21	Yes	***	-38.85 to -31.49
C90 vs C120	0,4950	0,6082	No	ns	-3.185 to 4.175
C90 vs G120	-43,67	53,65	Yes	***	-47.34 to -39.99
C90 vs GF120	-33,15	40,72	Yes	***	-36.83 to -29.47
C90 vs GR 120	-29,14	35,80	Yes	***	-32.82 to -25.46
G30 vs G60	-2,310	2,838	No	ns	-5.990 to 1.370
G30 vs G90	-6,450	7,924	Yes	***	-10.13 to -2.770
G30 vs C120	29,22	35,90	Yes	***	25.54 to 32.90
G30 vs G120	-14,94	18,36	Yes	***	-18.62 to -11.26
G30 vs GF120	-4,423	5,434	Yes	**	-8.103 to -0.7435
G30 vs GR 120	-0,4133	0,5078	No	ns	-4.093 to 3.266
G60 vs G90	-4,140	5,086	Yes	*	-7.820 to -0.4602
G60 vs C120	31,53	38,74	Yes	***	27.85 to 35.21
G60 vs G120	-12,63	15,52	Yes	***	-16.31 to -8.952
G60 vs GF120	-2,113	2,596	No	ns	-5.793 to 1.566
G60 vs GR 120	1,897	2,330	No	ns	-1.783 to 5.576
G90 vs C120	35,67	43,82	Yes	***	31.99 to 39.35
G90 vs G120	-8,492	10,43	Yes	***	-12.17 to -4.812
G90 vs GF120	2,027	2,490	No	ns	-1.653 to 5.706
G90 vs GR 120	6,037	7,417	Yes	***	2.357 to 9.716
C120 vs G120	-44,16	54,26	Yes	***	-47.84 to -40.48
C120 vs GF120	-33,64	41,33	Yes	***	-37.32 to -29.96
C120 vs GR 120	-29,63	36,41	Yes	***	-33.31 to -25.95
	10,52	12,92	Yes	***	6.839 to 14.20
G120 vs GR 120	14,53	17,85	Yes	***	10.85 to 18.21

GF120 vs GR 120	4,010	4,927	Yes	*	0.3302 to 7.690

Parameter	
Table Analyzed	IL2
One-way analysis of variance	
P value	P<0.0001
P value summary	***
Are means signif. different? (	Yes
Number of groups	8
F	240,7
R squared	0,9768
Bartlett's test for equal variances	
Bartlett's statistic (corrected)	70,83
P value	P<0.0001
P value summary	***
Do the variances differ signif.	Yes

ANOVA Table	SS	df	MS
Treatment (between columns	6993	7	999,0
Residual (within columns)	166,0	40	4,150
Total	7159	47	

Tukey's Multiple Comparison	Mean Diff.	q	Significant	Summary	95% CI of diff
C90 vs G30	-20,34	24,46	Yes	***	-24.10 to -16.58
C90 vs G60	-25,29	30,41	Yes	***	-29.05 to -21.53
C90 vs G90	-31,31	37,64	Yes	***	-35.07 to -27.55
C90 vs C120	0,1083	0,1303	No	ns	-3.652 to 3.868
C90 vs G120	-34,19	41,11	Yes	***	-37.95 to -30.43
C90 vs GF120	-22,13	26,60	Yes	***	-25.89 to -18.37
C90 vs GR 120	-21,68	26,07	Yes	***	-25.44 to -17.92
G30 vs G60	-4,943	5,944	Yes	**	-8.703 to -1.183
G30 vs G90	-10,96	13,18	Yes	***	-14.72 to -7.202
G30 vs C120	20,45	24,59	Yes	***	16.69 to 24.21
G30 vs G120	-13,84	16,65	Yes	***	-17.60 to -10.08
G30 vs GF120	-1,782	2,142	No	ns	-5.542 to 1.978
G30 vs GR 120	-1,340	1,611	No	ns	-5.100 to 2.420
G60 vs G90	-6,018	7,236	Yes	***	-9.778 to -2.258
G60 vs C120	25,40	30,54	Yes	***	21.64 to 29.16
G60 vs G120	-8,900	10,70	Yes	***	-12.66 to -5.140
G60 vs GF120	3,162	3,802	No	ns	-0.5983 to 6.922
G60 vs GR 120	3,603	4,333	No	ns	-0.1567 to 7.363
G90 vs C120	31,41	37,77	Yes	***	27.65 to 35.17
G90 vs G120	-2,882	3,465	No	ns	-6.642 to 0.8783
G90 vs GF120	9,180	11,04	Yes	***	5.420 to 12.94
G90 vs GR 120	9,622	11,57	Yes	***	5.862 to 13.38
C120 vs G120	-34,30	41,24	Yes	***	-38.06 to -30.54
C120 vs GF120	-22,23	26,73	Yes	***	-25.99 to -18.47
C120 vs GR 120	-21,79	26,20	Yes	***	-25.55 to -18.03
G120 vs GF120	12,06	14,50	Yes	***	8.302 to 15.82
G120 vs GR 120	12,50	15,03	Yes	***	8.743 to 16.26

GF120 vs GR 120	0,4417	0,5311	No	ns	-3.318 to 4.202

Parameter					
Table Analyzed	IL4				
One-way analysis of variance					
P value	P<0.0001				
P value summary	***				
Are means signif. different? (P <	Yes				
Number of groups	8				
F	179,7				
R squared	0,9692				
Bartlett's test for equal variances					
Bartlett's statistic (corrected)	66,46				
P value	P<0.0001				
P value summary	***				
Do the variances differ signif. (P	Yes				
ANOVA Table	SS	df	MS		
Treatment (between columns)	3746	7	535,1		
Residual (within columns)	119,1	40	2,978		
Total	3865	47			
Tukey's Multiple Comparison Tes	Mean Diff.	q	Significant	Summary	95% CI of diff
C90 vs G30	-15,80	22,43	Yes	***	-18.99 to -12.62
C90 vs G60	-18,79	26,67	Yes	***	-21.98 to -15.61
C90 vs G90	-22,95	32,58	Yes	***	-26.14 to -19.77
C90 vs C120	-0,01467	0,02082	No	ns	-3.200 to 3.171
C90 vs G120	-24,07	34,16	Yes	***	-27.25 to -20.88
C90 vs GF120	-15,17	21,54	Yes	***	-18.36 to -11.99
C90 vs GR 120	-19,04	27,03	Yes	***	-22.23 to -15.86
G30 vs G60	-2,992	4,246	No	ns	-6.177 to 0.1935
G30 vs G90	-7,150	10,15	Yes	***	-10.34 to -3.965
G30 vs C120	15,79	22,41	Yes	***	12.60 to 18.97
G30 vs G120	-8,268	11,74	Yes	***	-11.45 to -5.083
G30 vs GF120	0,6267	0,8895	No	ns	-2.559 to 3.812
G30 vs GR 120	-3,243	4,604	Yes	*	-6.429 to -0.05813
G60 vs G90	-4,158	5,902	Yes	**	-7.344 to -0.9731
G60 vs C120	18,78	26,65	Yes	***	15.59 to 21.96
G60 vs G120	-5,277	7,490	Yes	***	-8.462 to -2.091
G60 vs GF120	3,618	5,136	Yes	*	0.4331 to 6.804
G60 vs GR 120	-0,2517	0,3572	No	ns	-3.437 to 2.934
G90 vs C120	22,94	32,56	Yes	***	19.75 to 26.12
G90 vs G120	-1,118	1,587	No	ns	-4.304 to 2.067
G90 vs GF120	7,777	11,04	Yes	***	4.591 to 10.96
G90 vs GR 120	3,907	5,545	Yes	**	0.7215 to 7.092
C120 vs G120	-24,06	34,14	Yes	***	-27.24 to -20.87
C120 vs GF120	-15,16	21,52	Yes	***	-18.35 to -11.97
C120 vs GR 120	-19,03	27,01	Yes	***	-22.22 to -15.84
G120 vs GF120	8,895	12,63	Yes	***	5.710 to 12.08
G120 vs GR 120	5,025	7,132	Yes	***	1.840 to 8.210

GF120 vs GR 120	-3,870	5,493	Yes	**	-7.055 to -0.6848

Parameter	
Table Analyzed	IL8

One-way analysis of variance	
P value	P<0.0001
P value summary	***
Are means signif. different? (P	Yes
Number of groups	8
F	181,6
R squared	0,9695

Bartlett's test for equal variances	
Bartlett's statistic (corrected)	67,25
P value	P<0.0001
P value summary	***
Do the variances differ signif. (	Yes

ANOVA Table	SS	df	MS
Treatment (between columns)	5743	7	820,4
Residual (within columns)	180,7	40	4,518
Total	5924	47	

Tukey's Multiple Comparison T	Mean Diff.	q	Significant	Summary	95% CI of diff
C90 vs G30	-17,26	19,89	Yes	***	-21.19 to -13.34
C90 vs G60	-22,60	26,05	Yes	***	-26.52 to -18.68
C90 vs G90	-28,28	32,59	Yes	***	-32.21 to -24.36
C90 vs C120	0,004667	0,005378	No	ns	-3.918 to 3.928
C90 vs G120	-30,20	34,81	Yes	***	-34.13 to -26.28
C90 vs GF120	-16,05	18,50	Yes	***	-19.98 to -12.13
C90 vs GR 120	-8,928	10,29	Yes	***	-12.85 to -5.004
G30 vs G60	-5,338	6,152	Yes	**	-9.261 to -1.415
G30 vs G90	-11,02	12,70	Yes	***	-14.94 to -7.097
G30 vs C120	17,27	19,90	Yes	***	13.34 to 21.19
G30 vs G120	-12,94	14,91	Yes	***	-16.86 to -9.017
G30 vs GF120	1,208	1,392	No	ns	-2.715 to 5.131
G30 vs GR 120	8,335	9,605	Yes	***	4.412 to 12.26
G60 vs G90	-5,682	6,548	Yes	***	-9.605 to -1.759
G60 vs C120	22,61	26,05	Yes	***	18.68 to 26.53
G60 vs G120	-7,602	8,760	Yes	***	-11.52 to -3.679
G60 vs GF120	6,547	7,544	Yes	***	2.624 to 10.47
G60 vs GR 120	13,67	15,76	Yes	***	9.750 to 17.60
G90 vs C120	28,29	32,60	Yes	***	24.36 to 32.21
G90 vs G120	-1,920	2,213	No	ns	-5.843 to 2.003
G90 vs GF120	12,23	14,09	Yes	***	8.305 to 16.15
G90 vs GR 120	19,36	22,30	Yes	***	15.43 to 23.28
C120 vs G120	-30,21	34,81	Yes	***	-34.13 to -26.28
C120 vs GF120	-16,06	18,51	Yes	***	-19.98 to -12.14
C120 vs GR 120	-8,932	10,29	Yes	***	-12.86 to -5.009
G120 vs GF120	14,15	16,30	Yes	***	10.23 to 18.07
G120 vs GR 120	21,28	24,52	Yes	***	17.35 to 25.20

GF120 vs GR 120	7,127	8,213	Yes	***	3.204 to 11.05

Parameter	
Table Analyzed	IL10

One-way analysis of variance	
P value	P<0.0001
P value summary	***
Are means signif. different?	Yes
Number of groups	8
F	538,2
R squared	0,9895

Bartlett's test for equal variances	
Bartlett's statistic (corrected)	38,46
P value	P<0.0001
P value summary	***
Do the variances differ signif	Yes

ANOVA Table	SS	df	MS
Treatment (between column	4974	7	710,6
Residual (within columns)	52,82	40	1,320
Total	5027	47	

Tukey's Multiple Comparison	Mean Diff.	q	Significant	Summary	95% CI of diff
C90 vs G30	-12,83	27,34	Yes	***	-14.95 to -10.71
C90 vs G60	-18,19	38,78	Yes	***	-20.31 to -16.07
C90 vs G90	-19,04	40,59	Yes	***	-21.16 to -16.92
C90 vs C120	0,1415	0,3016	No	ns	-1.979 to 2.262
C90 vs G120	-19,80	42,20	Yes	***	-21.92 to -17.67
C90 vs GF120	-26,48	56,45	Yes	***	-28.60 to -24.36
C90 vs GR 120	-28,85	61,51	Yes	***	-30.97 to -26.73
G30 vs G60	-5,367	11,44	Yes	***	-7.488 to -3.246
G30 vs G90	-6,215	13,25	Yes	***	-8.336 to -4.094
G30 vs C120	12,97	27,64	Yes	***	10.85 to 15.09
G30 vs G120	-6,968	14,85	Yes	***	-9.089 to -4.847
G30 vs GF120	-13,65	29,10	Yes	***	-15.77 to -11.53
G30 vs GR 120	-16,03	34,16	Yes	***	-18.15 to -13.91
G60 vs G90	-0,8483	1,808	No	ns	-2.969 to 1.273
G60 vs C120	18,34	39,08	Yes	***	16.21 to 20.46
G60 vs G120	-1,602	3,414	No	ns	-3.723 to 0.5192
G60 vs GF120	-8,287	17,66	Yes	***	-10.41 to -6.166
G60 vs GR 120	-10,66	22,72	Yes	***	-12.78 to -8.539
G90 vs C120	19,18	40,89	Yes	***	17.06 to 21.30
G90 vs G120	-0,7533	1,606	No	ns	-2.874 to 1.368
G90 vs GF120	-7,438	15,86	Yes	***	-9.559 to -5.317
G90 vs GR 120	-9,812	20,92	Yes	***	-11.93 to -7.691
C120 vs G120	-19,94	42,50	Yes	***	-22.06 to -17.82
C120 vs GF120	-26,62	56,75	Yes	***	-28.74 to -24.50
C120 vs GR 120	-29,00	61,81	Yes	***	-31.12 to -26.87
G120 vs GF120	-6,685	14,25	Yes	***	-8.806 to -4.564
G120 vs GR 120	-9,058	19,31	Yes	***	-11.18 to -6.937

GF120 vs GR 120	-2,373	5,059	Yes	*	-4.494 to -0.2524

Parameter					
Table Analyzed	IL18				
One-way analysis of variance					
P value	P<0.0001				
P value summary	***				
Are means signif. different? (P < 0.05)	Yes				
Number of groups	8				
F	217,7				
R squared	0,9744				
Bartlett's test for equal variances					
Bartlett's statistic (corrected)	57,12				
P value	P<0.0001				
P value summary	***				
Do the variances differ signif. (P < 0.05)	Yes				
ANOVA Table	SS	df	MS		
Treatment (between columns)	4398	7	628,3		
Residual (within columns)	115,4	40	2,886		
Total	4514	47			
Tukey's Multiple Comparison Test	Mean Diff.	q	Significant	Summary	95% CI of diff
C90 vs G30	-7,216	10,40	Yes	***	-10.35 to -4.080
C90 vs G60	-14,60	21,05	Yes	***	-17.73 to -11.46
C90 vs G90	-23,90	34,46	Yes	***	-27.03 to -20.76
C90 vs C120	0,02567	0,03701	No	ns	-3.110 to 3.161
C90 vs G120	-28,36	40,89	Yes	***	-31.49 to -25.22
C90 vs GF120	-15,06	21,72	Yes	***	-18.20 to -11.93
C90 vs GR 120	-12,99	18,73	Yes	***	-16.12 to -9.854
G30 vs G60	-7,380	10,64	Yes	***	-10.52 to -4.245
G30 vs G90	-16,68	24,05	Yes	***	-19.82 to -13.55
G30 vs C120	7,242	10,44	Yes	***	4.106 to 10.38
G30 vs G120	-21,14	30,48	Yes	***	-24.28 to -18.00
G30 vs GF120	-7,847	11,31	Yes	***	-10.98 to -4.711
G30 vs GR 120	-5,773	8,325	Yes	***	-8.909 to -2.638
G60 vs G90	-9,302	13,41	Yes	***	-12.44 to -6.166
G60 vs C120	14,62	21,08	Yes	***	11.49 to 17.76
G60 vs G120	-13,76	19,84	Yes	***	-16.90 to -10.62
G60 vs GF120	-0,4667	0,6729	No	ns	-3.602 to 2.669
G60 vs GR 120	1,607	2,317	No	ns	-1.529 to 4.742
G90 vs C120	23,92	34,50	Yes	***	20.79 to 27.06
G90 vs G120	-4,458	6,429	Yes	**	-7.594 to -1.323
G90 vs GF120	8,835	12,74	Yes	***	5.700 to 11.97
G90 vs GR 120	10,91	15,73	Yes	***	7.773 to 14.04
C120 vs G120	-28,38	40,92	Yes	***	-31.52 to -25.25
C120 vs GF120	-15,09	21,76	Yes	***	-18.22 to -11.95
C120 vs GR 120	-13,01	18,77	Yes	***	-16.15 to -9.879
G120 vs GF120	13,29	19,17	Yes	***	10.16 to 16.43
G120 vs GR 120	15,37	22,16	Yes	***	12.23 to 18.50

GF120 vs GR 120	2,073	2,990	No	ns	-1.062 to 5.209

Parameter	
Table Analyzed	a-Klotho
One-way analysis of variance	
P value	P<0.0001
P value summary	***
Are means signif. different	Yes
Number of groups	8
F	317,5
R squared	0,9823
Bartlett's test for equal variances	
Bartlett's statistic (correcte	46,47
P value	P<0.0001
P value summary	***
Do the variances differ sig	Yes

ANOVA Table	SS	df	MS
Treatment (between colum	2964	7	423,4
Residual (within columns)	53,34	40	1,334
Total	3017	47	

Tukey's Multiple Comparis	Mean Diff.	q	Significant	Summary	95% CI of diff
C90 vs G30	-3,789	8,037	Yes	***	-5.921 to -1.658
C90 vs G60	-4,313	9,147	Yes	***	-6.444 to -2.181
C90 vs G90	-5,673	12,03	Yes	***	-7.804 to -3.541
C90 vs C120	0,02950	0,06257	No	ns	-2.102 to 2.161
C90 vs G120	-6,191	13,13	Yes	***	-8.322 to -4.059
C90 vs GF120	-15,06	31,95	Yes	***	-17.19 to -12.93
C90 vs GR 120	-24,67	52,33	Yes	***	-26.80 to -22.54
G30 vs G60	-0,5233	1,110	No	ns	-2.655 to 1.608
G30 vs G90	-1,883	3,995	No	ns	-4.015 to 0.2481
G30 vs C120	3,819	8,100	Yes	***	1.687 to 5.950
G30 vs G120	-2,402	5,094	Yes	*	-4.533 to -0.2702
G30 vs GF120	-11,27	23,91	Yes	***	-13.40 to -9.142
G30 vs GR 120	-20,88	44,29	Yes	***	-23.01 to -18.75
G60 vs G90	-1,360	2,885	No	ns	-3.491 to 0.7714
G60 vs C120	4,342	9,210	Yes	***	2.211 to 6.473
G60 vs G120	-1,878	3,984	No	ns	-4.010 to 0.2531
G60 vs GF120	-10,75	22,80	Yes	***	-12.88 to -8.619
G60 vs GR 120	-20,36	43,18	Yes	***	-22.49 to -18.23
G90 vs C120	5,702	12,09	Yes	***	3.571 to 7.833
G90 vs G120	-0,5183	1,099	No	ns	-2.650 to 1.613
G90 vs GF120	-9,390	19,92	Yes	***	-11.52 to -7.259
G90 vs GR 120	-19,00	40,30	Yes	***	-21.13 to -16.87
C120 vs G120	-6,220	13,19	Yes	***	-8.352 to -4.089
C120 vs GF120	-15,09	32,01	Yes	***	-17.22 to -12.96
C120 vs GR 120	-24,70	52,39	Yes	***	-26.83 to -22.57
G120 vs GF120	-8,872	18,82	Yes	***	-11.00 to -6.740
G120 vs GR 120	-18,48	39,20	Yes	***	-20.61 to -16.35

GF120 vs GR 120	-9,608	20,38	Yes	***	-11.74 to -7.477

Parameter	
Table Analyzed	KLF4
One-way analysis of variance	
P value	P<0.0001
P value summary	***
Are means signif. different? (P < 0.	Yes
Number of groups	8
F	498,2
R squared	0,9887
Bartlett's test for equal variances	
Bartlett's statistic (corrected)	49,95
P value	P<0.0001
P value summary	***
Do the variances differ signif. (P <	Yes

ANOVA Table	SS	df	MS
Treatment (between columns)	7775	7	1111
Residual (within columns)	89,18	40	2,230
Total	7864	47	

Tukey's Multiple Comparison Test	Mean Diff.	q	Significant	Summary	95% CI of diff
C90 vs G30	-22,96	37,67	Yes	***	-25.72 to -20.21
C90 vs G60	-24,75	40,61	Yes	***	-27.51 to -22.00
C90 vs G90	-32,36	53,09	Yes	***	-35.12 to -29.61
C90 vs C120	0,03350	0,05495	No	ns	-2.722 to 2.789
C90 vs G120	-33,78	55,42	Yes	***	-36.54 to -31.03
C90 vs GF120	-16,68	27,36	Yes	***	-19.44 to -13.92
C90 vs GR 120	-6,831	11,21	Yes	***	-9.587 to -4.075
G30 vs G60	-1,790	2,936	No	ns	-4.546 to 0.9660
G30 vs G90	-9,402	15,42	Yes	***	-12.16 to -6.646
G30 vs C120	23,00	37,72	Yes	***	20.24 to 25.75
G30 vs G120	-10,82	17,75	Yes	***	-13.58 to -8.066
G30 vs GF120	6,282	10,30	Yes	***	3.526 to 9.038
G30 vs GR 120	16,13	26,46	Yes	***	13.38 to 18.89
G60 vs G90	-7,612	12,49	Yes	***	-10.37 to -4.856
G60 vs C120	24,79	40,66	Yes	***	22.03 to 27.54
G60 vs G120	-9,032	14,82	Yes	***	-11.79 to -6.276
G60 vs GF120	8,072	13,24	Yes	***	5.316 to 10.83
G60 vs GR 120	17,92	29,40	Yes	***	15.17 to 20.68
G90 vs C120	32,40	53,15	Yes	***	29.64 to 35.15
G90 vs G120	-1,420	2,329	No	ns	-4.176 to 1.336
G90 vs GF120	15,68	25,73	Yes	***	12.93 to 18.44
G90 vs GR 120	25,53	41,89	Yes	***	22.78 to 28.29
C120 vs G120	-33,82	55,48	Yes	***	-36.57 to -31.06
C120 vs GF120	-16,71	27,42	Yes	***	-19.47 to -13.96
C120 vs GR 120	-6,864	11,26	Yes	***	-9.620 to -4.108
G120 vs GF120	17,10	28,06	Yes	***	14.35 to 19.86
G120 vs GR 120	26,95	44,22	Yes	***	24.20 to 29.71

GF120 vs GR 120	9,850	16,16	Yes	***	7.094 to 12.61

Parameter	
Table Analyzed	HOXA5

One-way analysis of variance	
P value	P<0.0001
P value summary	***
Are means signif. different? (	Yes
Number of groups	8
F	386,4
R squared	0,9854

Bartlett's test for equal variances	
Bartlett's statistic (corrected)	71,81
P value	P<0.0001
P value summary	***
Do the variances differ signif.	Yes

ANOVA Table	SS	df	MS
Treatment (between columns	7662	7	1095
Residual (within columns)	113,3	40	2,833
Total	7775	47	

Tukey's Multiple Comparison	Mean Diff.	q	Significant	Summary	95% CI of diff
C90 vs G30	-14,01	20,39	Yes	***	-17.12 to -10.90
C90 vs G60	-8,600	12,52	Yes	***	-11.71 to -5.493
C90 vs G90	-7,996	11,64	Yes	***	-11.10 to -4.890
C90 vs C120	0,1453	0,2115	No	ns	-2.961 to 3.252
C90 vs G120	-3,706	5,393	Yes	**	-6.812 to -0.5995
C90 vs GF120	-32,34	47,07	Yes	***	-35.45 to -29.23
C90 vs GR 120	-33,76	49,14	Yes	***	-36.87 to -30.65
G30 vs G60	5,412	7,876	Yes	***	2.305 to 8.518
G30 vs G90	6,015	8,754	Yes	***	2.909 to 9.121
G30 vs C120	14,16	20,60	Yes	***	11.05 to 17.26
G30 vs G120	10,31	15,00	Yes	***	7.199 to 13.41
G30 vs GF120	-18,33	26,68	Yes	***	-21.44 to -15.22
G30 vs GR 120	-19,75	28,74	Yes	***	-22.86 to -16.64
G60 vs G90	0,6033	0,8781	No	ns	-2.503 to 3.710
G60 vs C120	8,745	12,73	Yes	***	5.638 to 11.85
G60 vs G120	4,894	7,122	Yes	***	1.787 to 8.000
G60 vs GF120	-23,74	34,55	Yes	***	-26.85 to -20.64
G60 vs GR 120	-25,16	36,62	Yes	***	-28.27 to -22.06
G90 vs C120	8,142	11,85	Yes	***	5.035 to 11.25
G90 vs G120	4,290	6,244	Yes	**	1.184 to 7.397
G90 vs GF120	-24,35	35,43	Yes	***	-27.45 to -21.24
G90 vs GR 120	-25,77	37,50	Yes	***	-28.87 to -22.66
C120 vs G120	-3,851	5,605	Yes	**	-6.958 to -0.7448
C120 vs GF120	-32,49	47,28	Yes	***	-35.59 to -29.38
C120 vs GR 120	-33,91	49,35	Yes	***	-37.01 to -30.80
G120 vs GF120	-28,64	41,68	Yes	***	-31.74 to -25.53
G120 vs GR 120	-30,06	43,74	Yes	***	-33.16 to -26.95

GF120 vs GR 120	-1,420	2,067	No	ns	-4.526 to 1.686

Parameter	
Table Analyzed	NANOG

One-way analysis of variance	
P value	P<0.0001
P value summary	***
Are means signif. different?	Yes
Number of groups	8
F	216,4
R squared	0,9743

Bartlett's test for equal variances	
Bartlett's statistic (corrected	52,80
P value	P<0.0001
P value summary	***
Do the variances differ sign	Yes

ANOVA Table	SS	df	MS
Treatment (between colum	5751	7	821,5
Residual (within columns)	151,8	40	3,796
Total	5903	47	

Tukey's Multiple Compariso	Mean Diff.	q	Significant	Summary	95% CI of diff
C90 vs G30	-18,05	22,69	Yes	***	-21.64 to -14.45
C90 vs G60	-20,17	25,36	Yes	***	-23.77 to -16.57
C90 vs G90	-26,56	33,40	Yes	***	-30.16 to -22.97
C90 vs C120	0,06517	0,08193	No	ns	-3.531 to 3.661
C90 vs G120	-31,21	39,24	Yes	***	-34.80 to -27.61
C90 vs GF120	-14,59	18,34	Yes	***	-18.18 to -10.99
C90 vs GR 120	-6,332	7,962	Yes	***	-9.928 to -2.737
G30 vs G60	-2,123	2,670	No	ns	-5.719 to 1.472
G30 vs G90	-8,515	10,71	Yes	***	-12.11 to -4.919
G30 vs C120	18,11	22,77	Yes	***	14.52 to 21.71
G30 vs G120	-13,16	16,55	Yes	***	-16.76 to -9.566
G30 vs GF120	3,462	4,352	No	ns	-0.1341 to 7.057
G30 vs GR 120	11,72	14,73	Yes	***	8.119 to 15.31
G60 vs G90	-6,392	8,036	Yes	***	-9.987 to -2.796
G60 vs C120	20,24	25,44	Yes	***	16.64 to 23.83
G60 vs G120	-11,04	13,88	Yes	***	-14.63 to -7.443
G60 vs GF120	5,585	7,022	Yes	***	1.989 to 9.181
G60 vs GR 120	13,84	17,40	Yes	***	10.24 to 17.43
G90 vs C120	26,63	33,48	Yes	***	23.03 to 30.22
G90 vs G120	-4,647	5,842	Yes	**	-8.242 to -1.051
G90 vs GF120	11,98	15,06	Yes	***	8.381 to 15.57
G90 vs GR 120	20,23	25,44	Yes	***	16.63 to 23.83
C120 vs G120	-31,27	39,32	Yes	***	-34.87 to -27.68
C120 vs GF120	-14,65	18,42	Yes	***	-18.25 to -11.06
C120 vs GR 120	-6,398	8,044	Yes	***	-9.993 to -2.802
G120 vs GF120	16,62	20,90	Yes	***	13.03 to 20.22
G120 vs GR 120	24,88	31,28	Yes	***	21.28 to 28.47

GF120 vs GR 120	8,253	10,38	Yes	***	4.658 to 11.85

Parameter	
Table Analyzed	HIF1a

One-way analysis of variance	
P value	P<0.0001
P value summary	***
Are means signif. different? (	Yes
Number of groups	8
F	554,2
R squared	0,9898

Bartlett's test for equal variances	
Bartlett's statistic (corrected)	46,54
P value	P<0.0001
P value summary	***
Do the variances differ signif.	Yes

ANOVA Table	SS	df	MS
Treatment (between columns	7226	7	1032
Residual (within columns)	74,51	40	1,863
Total	7301	47	

Tukey's Multiple Comparison	Mean Diff.	q	Significant	Summary	95% CI of diff
C90 vs G30	-19,93	35,77	Yes	***	-22.45 to -17.41
C90 vs G60	-24,65	44,25	Yes	***	-27.17 to -22.14
C90 vs G90	-28,45	51,05	Yes	***	-30.96 to -25.93
C90 vs C120	0,0590	0,1059	No	ns	-2.460 to 2.578
C90 vs G120	-33,02	59,26	Yes	***	-35.54 to -30.50
C90 vs GF120	-13,50	24,23	Yes	***	-16.02 to -10.98
C90 vs GR 120	-3,663	6,574	Yes	***	-6.182 to -1.144
G30 vs G60	-4,723	8,477	Yes	***	-7.242 to -2.204
G30 vs G90	-8,515	15,28	Yes	***	-11.03 to -5.996
G30 vs C120	19,99	35,88	Yes	***	17.47 to 22.51
G30 vs G120	-13,09	23,49	Yes	***	-15.61 to -10.57
G30 vs GF120	6,433	11,55	Yes	***	3.914 to 8.952
G30 vs GR 120	16,27	29,20	Yes	***	13.75 to 18.79
G60 vs G90	-3,792	6,805	Yes	***	-6.311 to -1.273
G60 vs C120	24,71	44,35	Yes	***	22.19 to 27.23
G60 vs G120	-8,365	15,01	Yes	***	-10.88 to -5.846
G60 vs GF120	11,16	20,02	Yes	***	8.638 to 13.68
G60 vs GR 120	20,99	37,68	Yes	***	18.47 to 23.51
G90 vs C120	28,51	51,16	Yes	***	25.99 to 31.02
G90 vs G120	-4,573	8,208	Yes	***	-7.092 to -2.054
G90 vs GF120	14,95	26,83	Yes	***	12.43 to 17.47
G90 vs GR 120	24,78	44,48	Yes	***	22.26 to 27.30
C120 vs G120	-33,08	59,37	Yes	***	-35.60 to -30.56
C120 vs GF120	-13,56	24,33	Yes	***	-16.08 to -11.04
C120 vs GR 120	-3,722	6,680	Yes	***	-6.241 to -1.203
G120 vs GF120	19,52	35,04	Yes	***	17.00 to 22.04
G120 vs GR 120	29,36	52,69	Yes	***	26.84 to 31.88

GF120 vs GR 120	9,835	17,65	Yes	***	7.316 to 12.35
