# Supplementary material for: Myocardial Expression of Pluripotency, Longevity, and Proinflammatory Genes in the Context of Hypercholesterolemia and Statin Treatment
Source: J Clin Med. 2024 Mar 29;13(7):1994. doi: 10.3390/jcm13071994 (PMC11012955; doi:10.3390/jcm13071994)
Supplement: Supplementary file 1 [file jcm-13-01994-s001.zip › Supplemental Table S1. Primers.pdf]

Supplemental Table S1. Primers

Rabbit target gene	Forward primer	Reverse primer	Target size	NM
**β-actin**	CCATGTACGTGGCCATCCAG	TCTTCATGAGGTAGTCGGTCAGGTC	148	NM_001101683
**MYD88**	CCCTTTGTCTCTCGACTCTTGG	TACGAGAACAGCCACTGCCC	125	Trace Archive
**NF-κB**	ATGCCAATGCCCTCTTCGACT	CGTGACTTCCAGCAGATCCCT	122	Trace Archive
**CCL4**	GAGACCACCAGCCTCTGCTC	TCAGTTCAGTTCCAAGTCATCCAC	123	NM_001082196
**CCL20**	TATCGTGGGCTTCACACAGC	CCATTCCTTCTTCGGATCTGC	115	Trace Archive
**CCR2**	GGTTGCTGAGAAGCCTGACACGC	CAGGTCTGTATTCTTCAACAAGCCC TCG	125	Trace Archive
**IFN-β**	TCCAACTATGGCACGGAAGTCT	TTCTGGAGCTGTTGTGGTTCCT	133	XM_002707968
**IFN-γ**	TGCCAGGACACACTAACCAGAG	TGTCACTCTCCTCTTTCCAATTCC	127	NM_001081991
**IL-1β**	TTGAAGAAGAACCCGTCCTCTG	CTCATACGTGCCAGACAACACC	128	NM_001082201
**IL-2**	GCCCAAGAAGGTCACAGAATTG	TGCTGATTGATTCTCTGGTATTTCC	128	NM_001163180
**IL-4**	CGACATCATCCTACCCGAAGTC	CCTCTCTCTCGGTTGTGTTCTTG	122	NM_001163177
**IL-8**	CCACACCTTTCCATCCCAAAT	CTTCTGCACCCACTTTTCCTTG	122	NM_001082293
**IL-10**	CTTTGGCAGGGTGAAGACTTTC	ACTGGATCATCTCCGACAAGG	126	NM_001082045
**IL-18**	ACCAAGGACAGCAACCTGTGTT	ACAGAGAGGCTTACAGCCATGC	120	NM_001122940
**α-Klotho**	CAGCGACGGCTACAACAATG	AGCAGAGTTCGGCGTAATCC	296	Trace Archive
**KLF4**	ACGTACTCGCCTTGCTGATG	CAAAAACCCCGAATTGCCCG	151	XM_017347259.1
**HOXA5**	CATGCTCTTTGCCTCTCCGA	TTAGGGCAACGAGAACAGGG	248	Trace Archive
**NANOG**	TACCCAGGCTTCTACCCTGC	GGTTACTCCACGACTGGCTG	136	XM_002712762.1
**HIF1α**	TTTTGGCAGCAACGACACAG	GTGCAGGGTCAGCACTACTT	173	Trace Archive

*Myeloid differentiation primary response 88 (*MYD88*); Nuclear factor kappa-light-chain-enhancer of activated B cells (NF-κB); C-C chemokine ligand (CCL4); Chemokine (C-C motif) ligand 20 (CCL20); C-C chemokine receptor type 2 (CCR2); Interferon beta (IFN-β);* Interferon gamma (IFN-γ*); interleukin 1β (IL-1β); interleukin 2 (IL-2); interleukin 4 (IL-4); interleukin 8 (IL-8); interleukin 10 (IL-10); interleukin 18 (IL-18); krüppel-like factor 4 (KLF4); hypoxia-inducible factor 1-alpha (HIF1α)*
